# The Emergency Medicine Group Standardized Letter of Evaluation as a Workplace-based Assessment: The Validity Is in the Detail

**DOI:** 10.5811/westjem.2020.3.45077

**Published:** 2020-04-21

**Authors:** Jeffrey N. Love, Christopher I. Doty, Jessica L. Smith, Nicole M. Deiorio, Jaime Jordan, Michael W. Van Meter, Mary Ann Edens, Cullen B. Hegarty

**Affiliations:** * Georgetown University Hospital, Department of Emergency Medicine, Washington, District of Columbia; † University of Kentucky College of Medicine, Department of Emergency Medicine, Lexington, Kentucky; ‡ Alpert Medical School, Brown University, Department of Emergency Medicine, Providence, Rhode Island; § Virginia Commonwealth University School of Medicine, Department of Emergency Medicine, Richmond, Virginia; ¶ David Geffen School of Medicine, Harbor UCLA Medical Center, Department of Emergency Medicine, Torrance, California; || McGovern Medical School, University of Texas-Houston, Department of Emergency Medicine, Houston, Texas; # Louisiana State University School of Medicine-Shreveport, Department of Emergency Medicine, Shreveport, Louisiana; ** University of Minnesota-HealthPartners Institute/Region Hospital, Department of Emergency Medicine, Saint Paul, Minnesota

## Abstract

**Introduction:**

Interest is growing in specialty-specific assessments of student candidates based on clinical clerkship performance to assist in the selection process for postgraduate training. The most established and extensively used is the emergency medicine (EM) Standardized Letter of Evaluation (SLOE), serving as a substitute for the letter of recommendation. Typically developed by a program’s leadership, the group SLOE strives to provide a unified institutional perspective on performance. The group SLOE lacks guidelines to direct its development raising questions regarding the assessments, processes, and standardization programs employ. This study surveys EM programs to gather validity evidence regarding the inputs and processes involved in developing group SLOEs.

**Methods:**

A structured telephone interview was administered to assess the input data and processes employed by United States EM programs when generating group SLOEs.

**Results:**

With 156/178 (87.6%) of Accreditation Council of Graduate Medical Education-approved programs responding, 146 (93.6%) reported developing group SLOEs. Issues identified in development include the following: (1) 84.9% (124/146) of programs limit the consensus process by not employing rigorous methodology; (2) several stakeholder groups (nurses, patients) do not participate in candidate assessment placing final decisions at risk for construct under-representation; and (3) clinical shift assessments don’t reflect the task-specific expertise of each stakeholder group nor has the validity of each been assessed.

**Conclusion:**

Success of the group SLOE in its role as a summative workplace-based assessment is dependent upon valid input data and appropriate processes. This study of current program practices provides specific recommendations that would strengthen the validity arguments for the group SLOE.

## INTRODUCTION

Based on the challenge of selecting candidates whose performance and characteristics are a good fit, postgraduate programs are increasingly turning to specialty-specific assessments of clinical performance to determine who to interview. Although emergency medicine (EM) developed this approach in 1997, many specialties have recently either explored or initiated a similar tool: otolaryngology;^1^,^2^ dermatology;^3^ pediatrics;^4^ ophthalmology;^5^ internal medicine;^6^ plastic surgery;^7^ and general surgery.^8^ These assessments generally involve the development of a specialty-specific template. Authors are asked to complete the template, assessing clinical performance based on direct observation in predetermined competencies (eg, interpersonal skills, decision-making, etc) important to the practice of that specialty. Each competency is rated on a normative basis to serve the intended purpose of differentiating performance. The template provides a degree of standardization by creating a shared mental model of assessment.

According to Messick and others, all validity is construct validity consisting of five categories of evidence: content; response process; internal structure; relationship to other variables; and consequences.^9^–^11^ Originally developed for assessment by a single author based solely on that faculty member’s personal experience, early work demonstrated content-related validity^12^ that has been verified.^13^ Internal structure evidence has also been shown in the improved inter-rater reliability and discrimination of the Standardized Letter of Evaluation (SLOE) as compared to traditional narrative letters of recommendation that it has replaced.^12^,^14^ Finally, validity evidence of relations with other variables stems from a single study that the SLOE is one of the best predictors of clinical performance as a resident^15^

Although the SLOE is primarily an assessment of clinical performance, it has not previously been held to the standard of workplace-based assessments (WBA).^16^ Valid WBAs are based on a number of underlying tenets that reflect a global perspective on complex, multifaceted performance through “pixilation.” This process employs multisource assessments during a specified period in time to paint a picture of performance, understanding that it varies case-by-case based on factors related to the learner, patient, environment, and assessor.^17^–^21^ Appropriate development of WBAs includes the following:

1. Different perspectives on the same performance represents alternative, complimentary interpretations that are valid. As such, consensus must be reached regarding these varying perspectives to accurately reflect global performance.^20^–^22^
2. The input of all groups engaged in the provision of clinical care through 360° assessment based on direct observation.^19^,^22^,^23^
3. The use of assessment instruments that ask the right questions of assessors reflecting their task-specific expertise.^20^,^24^,^25^
4. An appropriate number of assessments from each expertise group to establish reliability.^26^,^27^
5. A balance of quantitative and qualitative performance data that capture the context-specific aspects of performance.^20^,^22^,^28^,

**Population Health Research Capsule**

What do we already know about this issue?

The group SLOE, an assessment of clinical performance, is the most important factor in determining which medical students to interview for postgraduate training in emergency medicine.

What was the research question?

To explore the inputs and processes in group SLOE development to evaluate its response process and internal structure validity.

What was the major finding of the study?

The inputs and processes employed by programs in group SLOE development are not well aligned with tenets of workplace assessments.

How does this improve population health?

Based on the findings of this study, expert consensus guidelines were developed and presented that would improve the validity of this summative, high stakes assessment.

**Figure 1:**
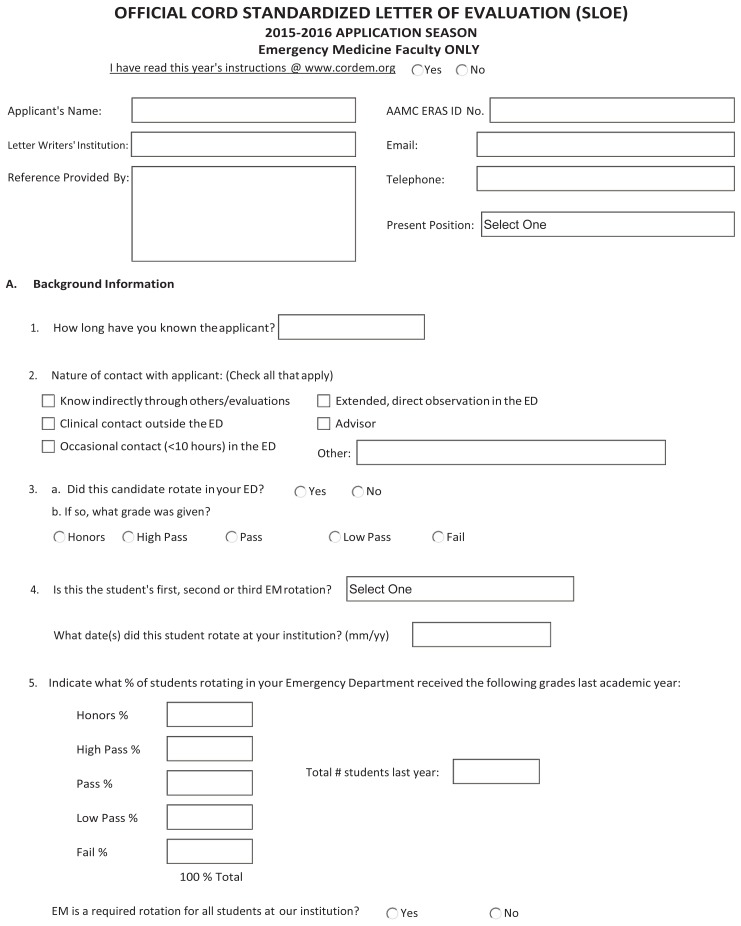

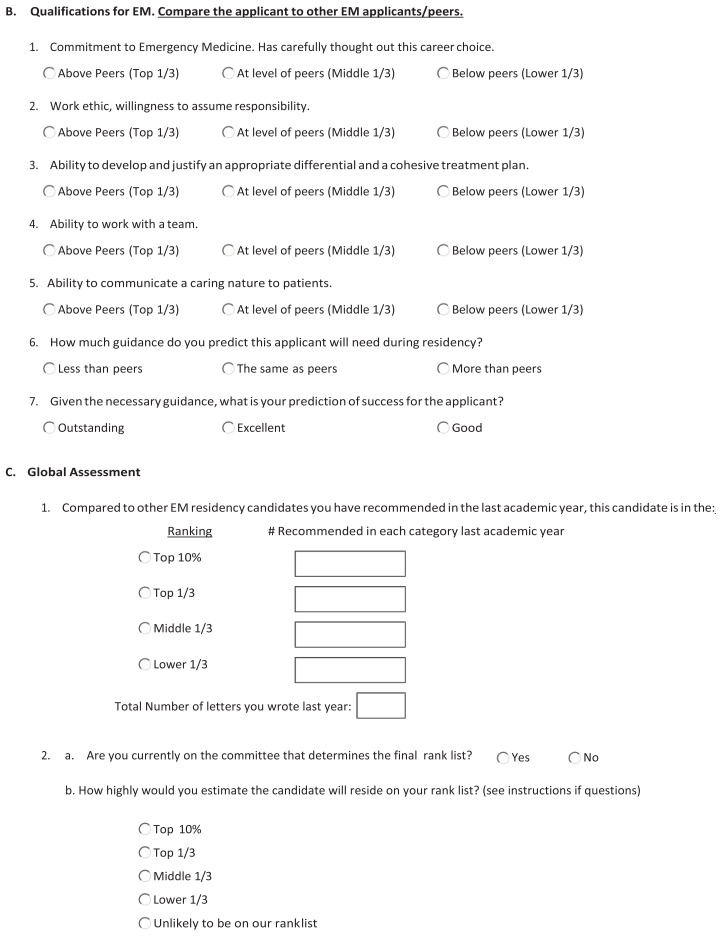

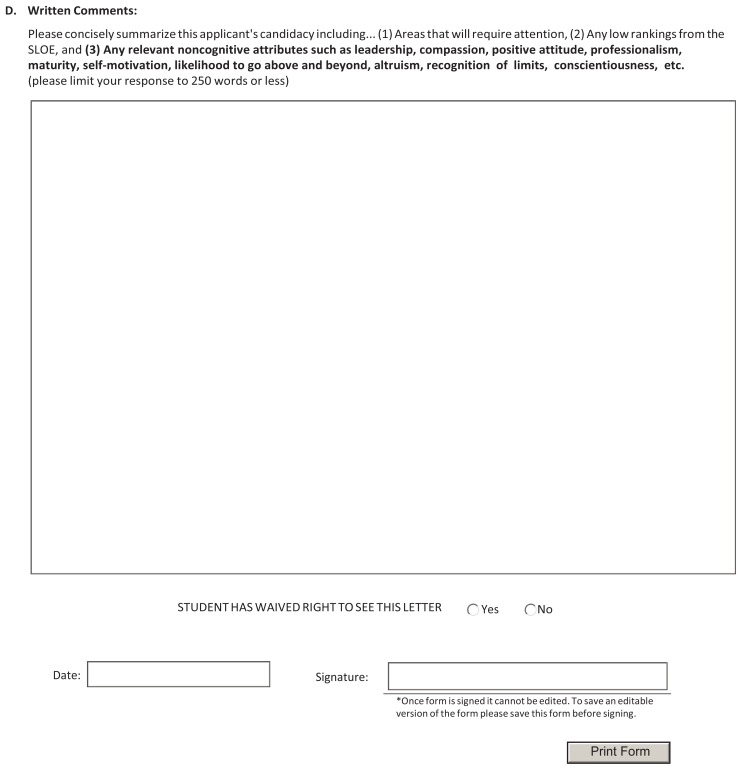
Standardized letter of evaluation template.

A fortuitous development from the single-author version, the group SLOE has become the preferred version of the SLOE by EM program directors (PD).^13^,^29^ As a summative assessment completed by departmental leadership, based on multisource feedback, the group SLOE should theoretically be less prone to individual bias and better positioned to provide a global perspective on clinical performance than the single- author version.^20^,^30^,^31^ Based largely on a variety of clinical assessments of performance, the group SLOE demonstrates internal structure evidence such as (1) committee member feels that his or her perspective is reflected in the final assessments,^32^ and (2) the group SLOE is more discriminating than the single-author version^13^ Although the single-author version has guidelines for completion (https://www.cordem.org/resources/residency-management/sloe/esloe/) and both versions are standardized based on the SLOE template (Figure 1), there are no guidelines in place that direct the multisource assessments and the process by which a group SLOE is drafted.^16^

As a high stakes-summative assessment, a strong validity argument is particularly important to all stakeholders of the group SLOE. The goal of this study was to explore the inputs and processes involved in group SLOE decision-making to assess its response process and internal structure validity arguments.

## METHODS

### Study Setting and Participants

A list of Accreditation Council for Graduate Medical Education (ACGME)-approved EM residencies was accessed on September 12, 2016, (https://apps.acgme.org/ads/Public/Programs/Search) identifying 178 unique programs. Potential participants were group SLOE authors, identified through review of group SLOEs that were submitted to the study team’s residency programs in the 2017 residency application cycle. One faculty member from each institution was invited to participate to avoid duplication, and all 178 programs were represented. Preference was given to the faculty member listed as the “contact author.” When a “contact author” could not be identified, we contacted that institution’s PD to determine the most appropriate faculty member to participate. Data were collected between February 17–June 2, 2017. The Georgetown School of Medicine Institional Review Board (IRB) determined this study protocol to be exempt from ongoing IRB review.

### Study Design

This was a cross-sectional study based on structured interviews. Study team members were assigned 18–19 programs based on geographic region. Standardized email invitations to participate were sent to assigned programs. In instances of no response after several attempts, study team members familiar with specific programs personnel reached out to those individuals to facilitate completion. Program representatives who agreed to participate were then scheduled for a telephone interview. It was estimated that participation would take approximately 20 minutes but frequently went longer based on the interviewee’s responses. Early in the formal data collection phase, the study team frequently conferred with each other to standardize management of unexpected responses or questions. The interviewer recorded participant responses by hand and then data were entered into a central database by each team member.

### Instrument and Decision Making

We initiated questionnaire development by a systematic review of the SLOE template to develop questions that ascertained input information (eg, assessors, assessment methods, and numbers) and the processes by which group SLOE committee decisions were made. To optimize content-related validity evidence of the questionnaire, we employed an iterative process. Final consensus of the eight-member investigation team included discussions regarding the degree to which inputs and processes were aligned with WBAs. Each member of this team had extensive experience as a SLOE author (mean 9.7 years) and leadership positions in resident training or medical student clerkships (mean 12.3 years). The final questionnaire consisted of multiple-choice questions. For each question there was a prompt for additional comments to clarify or expand on the answer provided (Appendix A).

Each item was read aloud and discussed among study authors to develop response process validity evidence of the questionnaire. Additionally, each author piloted the instrument with two or more experienced program leaders who were not involved in the study as author or participant (N = 20). As a result of this pilot, we changed a number of questions and developed a standardized script and strategy for the telephone interviews to improve the consistency of survey administration.

### Statistical Analysis

We calculated descriptive statistics including proportions and percentages for multiple-choice and completion items with numerical values using IBM SPSS Statistics for Macintosh, v. 20 (IBM Corporation, Armonk, NY). Free-response data were also collected for each question when appropriate.

## RESULTS

Of the 178 programs invited to participate, 156 responded to our inquiry (87.6%). Ten of 156 (6.4%) responding programs reported that they did not develop a group SLOE for candidates with the remaining 146 (93.6%) participating in the telephone interview that served as the basis for this study. Free-response data were insufficient to develop themes; instead they were used to raise issues and reinforce points regarding specific questions.

### Group SLOE Committees

The “contact authors” interviewed were 65.1% clerkship directors (95/146), 17.8% PDs (26/146), and the remaining 15.8% (23/146) consisted of associate PDs, vice chairs, or general faculty. Details of both the program’s and the “contact author’s” experience with the group SLOE are reported in Table 1. Group SLOE committees most commonly develop 16–45 SLOEs annually (96/146; 68.5%) with a range of 3–100. Table 2 lists the data identified as important to group SLOE decision-making and the relative importance of each.

“Shift cards” are assessments of clinical performance completed at the end of each clinical shift ; they may be structured, open-ended, or both. Excluding the two programs that did not use them, the average number of shift cards used per individual SLOE was program dependent (Table 3).

Shift cards are authored exclusively by faculty in 20.8% (30/144), by residents in 2.1% (3/144), and varying combinations of faculty and residents in 77.1% (111/144) of programs. Although the content of shift cards was not specifically queried, comments made by interviewees suggested variability across programs, with the majority using the seven questions regarding “qualifications for EM” from the SLOE template (Figure 1, Section B). There appears to be no difference between the templated shift cards completed by faculty and those by residents. Of the 32 programs that did not have residents complete shift cards, 11 collected clinical observations of student performance based on experiences with the teaching resident, pairing the candidate with a single resident for assessment, or formal meetings with residents monthly to obtain feedback.

### Group SLOE Process

When asked which one of the following processes comes closest to what is used in developing programs’ group SLOEs, “contact authors/spokesmen” responded: (1) One faculty leader reviews the data, generates the content and makes the decisions: 20.6% (30/146), (2) Two faculty leaders have these responsibilities: 47.9% (70/146), (3) Three or more committee members divide responsibilities which are then assembled: 16.4% (24/146), and (4) Three or more committee members come together to generate the entire content by consensus: 15.1% (22/146). Regardless of the process used, many programs added that they shared the draft version for comment by others. In instances where one faculty leader was responsible for the entire process (n = 30), 20 were clerkship directors, five PDs, and the remaining five had various other roles in the program.

When asked about how programs go about rating candidates on the seven qualifications/competencies for EM (Figure 1, Section B), 63.7% (93/146) reported using gestalt judgment, 27.4% (40/146) a combination of gestalt and a more formal approach, and 8.2% (12/146) based these assessments on a formal approach only. When a structured approach was used, 45/52 instances involved specific ratings requested on shift cards mirroring questions and ratings on section B of the group SLOE template (Figure 1).

In developing the written comments section for the group SLOE (Figure 1, Section D), authors use the following sources of information: 98.6% (144/146)-themes developed from shift cards, 97.9% (143/146)-first hand clinical experience, 72.6% (106/146)-verbatim comments from shift cards, 64.4% (94/146)-advising meeting between faculty and student and 12.3% (18/146)-suggestions made by the student. In the latter instance, 13/18 added that they would use such suggestions only if they were consistent with the authors’ experience.

### Work Group Process

The study authors reviewed the data and through iterative discussion came to a consensus on five key recommendations, which are summarized in Table 4.

## DISCUSSION

According to Johnston, “truth” in WBAs is a “matter of consensus among assessors who arrive at judgments on performance that are as informed and sophisticated as can be for that point in time.”^31^ To be an effective consensus process, committee decisions should follow established methodology. One such example is the nominal group technique.^33^–^35^ Active discussion that includes a diversity of faculty perspectives (eg, PDs, clerkship directors, other faculty) possessing firsthand clinical experience with candidates is important to final decisions. Several faculty members simply approving a final assessment authored by one or two faculty members does not constitute a rigorous consensus effort despite the use of multisource feedback in those decisions. In this study, only 15.1% of the programs developed group SLOE content by consensus-building with three or more members at the table. Recommendations #1 and #2 in Table 4 reflect the work group’s attention to this concept.

Expertise is the sine qua non of assessment, placing faculty squarely at the center of group SLOE development.^25^,^36^,^37^ Consistent with this principle, the single most important factor in group SLOE decision-making is shift cards with faculty participating in these assessments in 96.6% of programs. The second most important factor in group SLOE decision-making is clinical experience of committee members who bring their perspective to deliberations regarding the candidate being assessed. Experienced clinicians are less prone to the cognitive bias of the halo effect, are better judges of specific domains of complex performance, and are more appropriate judges of summative measures of global performance.^20^,^36^

While EM programs demonstrate an understanding of the importance of resident assessment of students when developing group SLOEs, this study reveals that many programs use faculty and resident assessment interchangeably to a varying degree (77.1%) when completing shift cards. Not only are residents less able to assess complex performance such as sophistication in developing evaluation/treatment plans or global performance, several studies suggest that they are also prone to leniency bias relative to faculty when assessing the same performance.^38^–^41^ This inter-institutional variability in who completes shift cards likely limits the equivalence and validity of group SLOE assessments. In addition, only 9.6% of programs included nursing assessments and none reported using patient feedback when developing group SLOEs, thereby risking construct under-representation in areas such as interpersonal skills and ability to work in teams (Recommendation #3, Table 4).

The validity of the group SLOE assessments would be improved by the development of simple, brief shift cards specific to each assessment group with questions reflecting the task specific expertise of each^19^ (Recommendation #3, Table 4). Until reliability numbers can be established, an average of one shift card per shift (~10–18/month) from each assessment group appears to be a practical and sufficient sampling size to minimize the risk of sampling error.^26^,^27^

Prior work shows that EM PDs find value in each of the seven SLOE questions regarding “qualifications for training in the specialty” (Figure 1, Section B).^13^ Nonetheless, in this study multiple respondents voiced a concern about the high degree of intercorrelation between scoring these seven competencies for any given candidate. This may be in part due to research that suggests only two dominant domains are consistent determinants of performance: interpersonal skills/humanism, and knowledge/problem-solving.^42^–^45^ Another potential cause of this lack of discrimination across qualifications may result from 67% of programs reporting that they used gestalt alone in determining the normative rating and an additional 27.4% used gestalt in combination with some standardized scoring on shift cards. Such an approach is prone to halo bias and appears to have limited value. Comments from survey participants suggest difficulty in obtaining stratified ratings from shift cards, which appear to be a current limitation of these assessment tools. A potential solution to this issue is provided by studies on clinical assessments of performance with construct-aligned scales demonstrating improved agreement and discrimination in assessments.^19^,^46^ Such scales use construct-based anchors that reflect performance of increasing sophistication and independence and are consistent with how assessors view development. Both evaluation of the number of performance domains and the development of instruments as suggested by Crossley et al^46^ may be helpful in developing appropriate assessments to assist in group SLOE decision-making regarding “qualifications for EM training” (Recommendation #4, Table 4).

The SLOE, consistent with tenets of summative WBAs, was designed to balance quantitative performance data and qualitative written comments that capture the context-specific aspects of performance.^20^,^22^,^28^ In reviewing the means by which group SLOE committees create a candidate’s written comments, they appear aligned with these tenets as long as development includes the same active consensus-building process necessary in other aspects of the group SLOE.

## STUDY STRENGTHS AND LIMITATIONS

With a response rate of 87.6%, this study does not appear to suffer from coverage, sampling, or nonresponse errors that are potential limitations of any survey. The educational leadership in emergency medicine is a relatively small community. This increases the likelihood that at least some interviewees knew the interviewers they were talking to introducting the potential for bias in the answers provided. In addition, an essential assumption of any valid assessment of clinical performance is that it is based on direct observation, which this study assumed but did not evaluate. These issues should be considered when interpreting our data.

## CONCLUSION/FUTURE DIRECTIONS

Validity is always an argument regarding degree; it is never absolute. The greater the validity evidence available, the stronger the argument. The EM group SLOE is a high stakes, summative assessment. As such, it must be held to a high standard by rigorous methodology, assessing validity evidence and enacting needed change to the template, inputs, and processes related to group SLOE development.

By aligning the process of group SLOE construction with tenets of WBAs, this study representing the practice of EM programs provides specific insights regarding initiatives and related studies that would improve the response process and internal structure validity evidence of group SLOE assessments. Substantial progress on these validity determinations would set the stage for standardization across programs (Recommendation #5, Table 4). In the meantime, programs should be mindful of the issues elucidated by this study when developing and interpreting group SLOEs.

## Supplementary Information





## Figures and Tables

**Table 1:** Emergency medicine program’s and contact author’s experience with standardized letters of evaluation (SLOE).

Question	0 years N=146	1–5 years N=146	6–10 years N=146	11–15 years N=146	>15 years N=146
Program’s experience with group SLOEs	X	74 (50.7%)	55 (37.7%)	11 (7.5%)	6 (4%)
Contact author’s experience with single-author SLOEs	48 (32.9%)	49 (33.6%)	34 (23.3%)	11 (7.5%)	4 (2.7%)
	≤ 1 year	2–3 years	4–6 years	6–9 years	> 9 years
Contact author’s experience with group SLOEs	14 (9.6%)	56 (38.4%)	45 (30.8%)	19 (13.0%)	12 (8.2%)

**Table 2:** Inputs to the group standardized letter of evaluation (SLOE) and their relative importance.

Inputs	Important in the overall decision making regarding the group SLOE (percentage of total agreeing to significance) N=146	Mean relative importance overall (3-very, 2-moderately, 1-minor importance)
Shift cards	144 (98.6%)	2.8
Firsthand clinical experience of group SLOE committee members	143 (97.9%)	2.4
Resident assessments	125 (85.6%)	2.1
Personal traits & information	114 (78.1%)	1.3
EM shelf exam	96 (65.8%)	1.2
Simulation	79 (54.1%)	1.5
USMLE	63 (43.2%)	1.2
Core clinical rotation grades	27 (18.5%)	0.3
Formal nurses’ assessments	14 (9.6%)	0.2
Medical school class rank	9 (6.2%)	0.1

*EM*, emergency medicine; *USMLE*, United States Medical Licensing Examination.

**Table 3:** Average number of shift cards used by programs to develop each standardized letter of evaluation.

Number of shift cards	Percentage of programs N=144
1–5	8 (5.6%)
6–10	53 (36.8%)
11–15	63 (43.8%)
16–20	16 (11.1%)
>20	4 (2.8%)

**Table 4:** Recommendations for improving the response process and internal structure validity of the group standard letter of evaluation (SLOE).

Group SLOE committee deliberations should be based on established consensus methodology. To promote a global perspective on performance, group SLOE committee membership should be broad and inclusive (with clerkship and program leadership as a requirement). All stakeholder groups involved in the provision of care (ie, faculty, residents, nurses, and patients) should participate in the assessment of student performance based on direct observation in the clinical environment. Unique shift assessments, reflecting the task expertise of each stakeholder group, need to be developed and validated. Guidelines should be established as standards are developed to guide programs in the assessment data and the processes by which group SLOEs are developed.
