# Supplementary material for: The Emergency Medicine Group Standardized Letter of Evaluation as a Workplace-based Assessment: The Validity Is in the Detail
Source: West J Emerg Med. 2020 Apr 21;21(3):600–9. doi: 10.5811/westjem.2020.3.45077 (PMC7234706; doi:10.5811/westjem.2020.3.45077)
Supplement: Supplementary file 1 [file wjem-21-600-g001.pdf]

OFFICIAL CORD STANDARDIZED LETTER OF EVALUATION (SLOE)

2015-2016 APPLICATION SEASON

Emergency Medicine Faculty ONLY

I have read this year's instructions @ www.cordem.org ◯ Yes ◯ No

Applicant's Name: ____________ AAMC ERAS ID No. ____________

Letter Writers' Institution: ____________ Email: ____________

Reference Provided By: ____________ Telephone: ____________

Present Position: Select One

A. Background Information

1. How long have you known the applicant? ____________

2. Nature of contact with applicant: (Check all that apply)

☐ Know indirectly through others/evaluations ☐ Extended, direct observation in the ED

☐ Clinical contact outside the ED ☐ Advisor

☐ Occasional contact (<10 hours) in the ED Other: ____________

3. a. Did this candidate rotate in your ED? ◯ Yes ◯ No

b. If so, what grade was given?

◯ Honors ◯ High Pass ◯ Pass ◯ Low Pass ◯ Fail

4. Is this the student's first, second or third EM rotation? Select One

What date(s) did this student rotate at your institution? (mm/yy) ____________

5. Indicate what % of students rotating in your Emergency Department received the following grades last academic year:

Honors % ____________

High Pass % ____________

Pass % ____________

Low Pass % ____________

Fail % ____________

100 % Total

Total # students last year: ____________

EM is a required rotation for all students at our institution? ◯ Yes ◯ No

B. Qualifications for EM. <u>Compare the applicant to other EM applicants/peers.</u>

1. Commitment to Emergency Medicine. Has carefully thought out this career choice.

   ◯ Above Peers (Top 1/3) ◯ At level of peers (Middle 1/3) ◯ Below peers (Lower 1/3)

2. Work ethic, willingness to assume responsibility.

   ◯ Above Peers (Top 1/3) ◯ At level of peers (Middle 1/3) ◯ Below peers (Lower 1/3)

3. Ability to develop and justify an appropriate differential and a cohesive treatment plan.

   ◯ Above Peers (Top 1/3) ◯ At level of peers (Middle 1/3) ◯ Below peers (Lower 1/3)

4. Ability to work with a team.

   ◯ Above Peers (Top 1/3) ◯ At level of peers (Middle 1/3) ◯ Below peers (Lower 1/3)

5. Ability to communicate a caring nature to patients.

   ◯ Above Peers (Top 1/3) ◯ At level of peers (Middle 1/3) ◯ Below peers (Lower 1/3)

6. How much guidance do you predict this applicant will need during residency?

   ◯ Less than peers ◯ The same as peers ◯ More than peers

7. Given the necessary guidance, what is your prediction of success for the applicant?

   ◯ Outstanding ◯ Excellent ◯ Good

C. Global Assessment

1. Compared to other EM residency candidates you have recommended in the last academic year, this candidate is in the:

Ranking	# Recommended in each category last academic year
◯ Top 10%	
◯ Top 1/3	
◯ Middle 1/3	
◯ Lower 1/3	

Total Number of letters you wrote last year: ____

2. a. Are you currently on the committee that determines the final rank list? ◯ Yes ◯ No

   b. How highly would you estimate the candidate will reside on your rank list? (see instructions if questions)

   ◯ Top 10%
   ◯ Top 1/3
   ◯ Middle 1/3
   ◯ Lower 1/3
   ◯ Unlikely to be on our ranklist

D. Written Comments:

Please concisely summarize this applicant's candidacy including... (1) Areas that will require attention, (2) Any low rankings from the SLOE, and **(3) Any relevant noncognitive attributes such as leadership, compassion, positive attitude, professionalism, maturity, self-motivation, likelihood to go above and beyond, altruism, recognition of limits, conscientiousness, etc.** (please limit your response to 250 words or less)

STUDENT HAS WAIVED RIGHT TO SEE THIS LETTER ◯ Yes ◯ No

Date:

Signature:

*Once form is signed it cannot be edited. To save an editable version of the form please save this form before signing.

Print Form
