# Supplementary material for: The Emergency Medicine Group Standardized Letter of Evaluation as a Workplace-based Assessment: The Validity Is in the Detail
Source: West J Emerg Med. 2020 Apr 21;21(3):600–9. doi: 10.5811/westjem.2020.3.45077 (PMC7234706; doi:10.5811/westjem.2020.3.45077)
Supplement: Supplementary file 2 [file wjem-21-600-s001.docx]

**Appendix A**

The Emergency Medicine Group Standardized Letter of Evaluation:

What’s the Standard?

1. Geographic location of program (Use the AAMCs determination of region)? ( Page 19 at this link: <https://www.aamc.org/download/164942/data/osr_handbook.pdf> minus Canada). In the study we will answer this question for participants and tell them what region they are in.
2. Northeastern
3. Southern
4. Central
5. Western
6. As of the 2016-2017 interview cycle, does your program write a “Group SLOE” ? Don’t define Group SLOE for them, if they say yes-gather the information.
7. If Yes, go on to complete the survey
8. If No, ask why your program does not and list in the space below. After completing this answer the survey for this program is complete.
9. How many years do you estimate that your Program has been completing Group SLOEs?
10. How many years of personal experience do you (the contact person) have as a SLOE author (Both single author and Group SLOE/SLORs)?
11. Single Author:
12. Group Author:
13. How many Group SLOEs did your institution write this year?
14. Do individual faculty members in your department complete single-author SLOEs?
15. Yes
16. No
17. Do any members of you Group SLOE Committee also write Single Author SLOEs
18. Yes
19. No
20. Who participates in authoring and editing your Group SLOE (Group SLOE Committee)? (Numbers of individuals in each category….Mark all relevant titles if a single author holds more than one position)
21. Departmental/Academic Chair
22. Vice Chair of Academic Affairs /Education
23. Program Director
24. Associate/Assistant Program Director(s)
25. Clerkship Director(s)
26. Associate/Assistant Clerkship Directors (s)
27. Other Faculty (please list the position of all others on the committee)
28. Please select **the one most applicable** method for creating your Group SLOE? (You may have to work with them a little to find the best fit)
29. One faculty leader reviews that available data, generates the content (rankings & written comments) and makes the decisions related to the group SLOE.
30. One lead author generates the content(rankings & written comments) which is then reviewed by two or more other Group SLOE authors.
31. Several Group SLOE authors individually generate some portion of the content (rankings & written comments) which are then reviewed by all of the Group SLOE authors
32. All of the Group SLOE authors generate the content (rankings & written comments) together as a group without any one person individually responsible for creating SLOE content.
33. Other (Please specify)
34. We are trying to get a sense of gender diversity among Group SLOE authors. Could you share with us the number of males and females on your Group SLOE Committee?
35. Male (number)
36. Female (number)
37. Do you use formal end of shift evaluations otherwise known as shift cards when developing your Group SLOE
38. Yes (Go to Question #12)
39. No (Go to Question #14)
40. Who authors the end of shift evaluations used to develop your Group SLOE?
41. Exclusively Faculty
42. Exclusively Residents
43. Both residents and faculty
44. Please note any additional authors In this space
45. Please estimate the average number of shift cards/evaluations per applicant when developing the Group SLOEs

- 1-5
- 6-10
- 11-15
- 16-20
- 21 or more

1. Which of the following resources influence your Group SLOE?
2. End of shift evaluations
3. Yes (Go to Question A1)
4. No (Go to Question B)
5. Question A1: What is the relative importance of end of shift evaluations in Group SLOE **overall decision making**?

(Less important) 1 2 3 (very important)

1. Personal clinical experience of the Group Sloe Authors?
2. Yes (Go to question B1)
3. No (Go to question C)

- Question B1: What is the relative importance of author’s experience in Group SLOE **overall decision making**?

(Less important) 1 2 3 (very important)

1. **Formal** evaluations from **residents** **based on clinical experience (e.g EM competencies: interpersonal skills, commitment to EM, Ability to develop a differential & cohesive treatment plan, etc)** with candidates other than shift cards influence on your Group SLOE?
2. Yes (Go to question C1)
3. No (Go to question D)
4. Question C1: What is the relative importance of formal resident evaluation in Group SLOE **overall decision making?**

(Less important) 1 2 3 (very important)

1. **Formal** evaluations from **nurses** based on clinical experience with candidates?
2. Yes (Go to Question D1)
3. No (Go to Question E)
4. Question D1: What is the relative importance of formal resident evaluations in Group SLOE **overall decision making**?

(Less important) 1 2 3 (very important)

1. Shelf exam grade for emergency medicine rotation?
2. Yes (Go to Question E1)
3. No (Go to Question F)

- Question E1: What is the relative importance of the shelf exam grade on the EM Rotation in Group SLOE **overall decision making**?

(Less important) 1 2 3 (very important)

1. Performance on 3^rd^ year core clinical rotations (IM, Surgery, OB, Peds, etc)?
2. Yes (Go to Question F1)
3. No (Go to Question G)
4. Question E1: What is the relative importance of 3^rd^ year core clinical performance in Group SLOE **overall decision making**?

(Less important) 1 2 3 (very important)

1. Assessments from simulation performance?
2. Yes (Go to Question G1)
3. No (Go to Question H)
4. Question F1: What is the relative importance of simulation performance in Group SLOE **overall decision making**?

(Less important) 1 2 3 (very important)

1. USMLEs?
2. Yes (Go to Question H1)
3. No (Go to Question I)
4. Question H1: What is the relative importance of the UMSLEs in Group SLOE **overall decision making**?

(Less important) 1 2 3 (very important)

1. Medical school class rank?
2. Yes (Go to Question I1)
3. No (Go to Question J)
4. Question I1: What is the relative importance of medical school rank in Group SLOE **overall decision making**?

(Less important) 1 2 3 (very important)

1. Non-Academic information (e.g. past experiences, service, personal statement, interests, leadership activities)?
2. Yes (Go to Question J1)
3. No (Go to Question K)
4. Question J1: What is the relative importance of formal resident evaluations in Group SLOE **overall decision making**?

(Less important) 1 2 3 (very important)

1. Other (Please specify)

1. Have them look at the SLOE template for this one: Regarding Section B (Question B1-B7)-Qualifications for EM, “Do you have some standardized process for formally assessing each of these areas, or is this based on the groups “gestalt” or is it some combination? If they provide a standardized process or combination please have them explain and note in the space below
2. Have them look at the SLOE template for this one: When considering the two “Global Assessment” questions, C1 (“Compared to other EM Residency Candidates you have recommended in the last academic year, this candidate is in the top10/top/middle/lower third?” and C2 (“How highly would you estimate the candidate will reside on your rank list”) do feel that these two questions provide……….
3. Different information (If selected have them move on to Question #17)
4. The same information (if selected skip to Question 18)
5. If these two questions provide different information, **please describe how your Group SLOE committee treats the two questions differently?** (Open ended question with free form data below. This is a poorly defined issue so give them space to explore this one but don’t provide them with suggestions).
6. C1: Compared to other EM Residency Candidates you have recommended….
7. C2: How high on your rank list will this candidate reside?
8. When developing the content for the written comment section (Section D) which of the following do you use.
9. Verbatim comments taken from shift cards/evaluations or the equivalent

Yes No

1. Themes developed from comments taken from shift cards

Yes No

1. First-hand clinical experiences from Group SLOE Committee members

Yes No

1. Knowledge of applicants beyond their clinical performance

Yes No

1. Advising meeting between faculty and student

Yes No

1. Suggestions made by the student (If they answer yes, ask what is the context of suggestions made by students)

Yes No

1. Other (Please note specifics)
2. To what degree do you share the specific contents of the Group SLOE with individual candidates? If answering C, D or E please share what information is shared
3. Not at all
4. Rarely
5. Occasionally
6. Frequently
7. Always.
8. Other (Please explain if selected):
